# Cardiac Arrest During Exertion as a Presentation of Undiagnosed Kawasaki Disease: A Case Report

**DOI:** 10.3390/jcm13216380

**Published:** 2024-10-24

**Authors:** Justyna Zamojska, Piotr Kędziora, Agnieszka Januś, Krzysztof Kaczmarek, Elżbieta Smolewska

**Affiliations:** 1 Department of Pediatric Cardiology and Rheumatology, Medical University of Lodz, 91-738 Lodz, Poland; piotr.kedziora.dr@wp.pl (P.K.); daniluk.agnieszka1@gmail.com (A.J.); elzbieta.smolewska@umed.lodz.pl (E.S.); 2 Department of Electrocardiology, Medical University of Lodz, 92-213 Lodz, Poland; krzysztof.kaczmarek@umed.lodz.pl

**Keywords:** Kawasaki disease, coronary aneurysm, cardiac arrest, cardiac arrhythmias, electrocardiology, subcutaneous cardioverter defibrillator

## Abstract

**Background:** Kawasaki Disease (KD) is self-limited vasculitis, the main consequence of which may be involvement of the coronary arteries, especially in patients without treatment. It is estimated that coronary artery aneurysms occur in 15% to 25% of untreated children. Patients with coronary aneurysms may remain asymptomatic for years. The first symptom may be life-threatening sudden cardiac arrest or myocardial ischaemia. **Methods:** We report a case of a 17-year-old boy with an insignificant past medical history who presented with sudden cardiac arrest. **Results:** During diagnostics, channelopathies, structural heart defects, drug abuse, and myocardial infarction were excluded. The patient underwent coronary angiography, confirmed by CT angiogram of the coronary vessels, which revealed most likely ruptured, clotted, well-calcified aneurysm of the left anterior descending artery (LAD) with collateral circulation, probably a consequence of untreated Kawasaki disease in early childhood. **Conclusions:** Complications of KD should be considered in the differential diagnosis of sudden cardiac arrest, especially in a young person.

## 1. Introduction

Kawasaki disease (KD) is a syndrome of unknown cause, acute, self-limited vasculitis of small and medium-sized vessels that occurs mainly in children between 6 months and 5 years of age [1,2,3].

The disease is the leading cause of acquired heart disease in children in developed countries and the diagnosis of its typical form is made on the basis of clinical criteria such as the presence of fever lasting five or more days, accompanied by four out of five of symptoms: lymphadenopathy (≥15 mm diameter; typically unilateral); non-purulent bilateral conjunctivitis; oral changes such as reddening of the lips, diffuse inflammation of oral and pharyngeal mucosae and strawberry tongue; polymorphic rash; and sole and/or palm erythema with later skin desquamation [2,4,5].

Although the disease has been known for over 50 years, its aetiology is still unclear. Recent research also highlights the role of genetic predisposition in the development of the disease [6].

Children who do not fulfil the complete diagnostic criteria for KD are described as having incomplete or atypical KD. These patients may still be at risk for coronary artery abnormalities [7,8].

There are no pathognomonic laboratory tests for Kawasaki disease; however, in most cases, C-reactive protein (CRP) ≥ 3.0 mg/dL and/or erythrocyte sedimentation rate (ESR) ≥ 40 mm/h are present. Laboratory findings in patients with abnormalities in coronary arteries in echocardiographic examinations, which can help in the diagnosis of the disease in the absence of typical symptoms, are at least three out of six: anaemia, platelet count ≥ 450 g/L after the seventh day of fever, albumin concentration ≤ 3.0 g/dL, white blood cell count (WBC) ≥ 15.0 g/L, increased alanine transaminase, and, in urine, presence of ≥10 white blood cells/high powered field [2].

The first-line treatment for Kawasaki disease involves intravenous administration of immunoglobulins (IVIGs) at a dosage of 2 g/kg in combination with acetylsalicylic acid (ASA). The dosage of ASA, which varies between low (3–5 mg/kg), moderate (30–50 mg/kg), and high (80–100 mg/kg), is continued for 48 to 72 h until the patient’s fever subsides. US guidelines recommend both moderate and high doses of ASA. In most guidelines, ASA in low doses should be used only in maintenance therapy to avoid thrombotic complications and continued until the coronary lesions have resolved. Corticosteroids as a first-line treatment remain controversial; they are recommended only in patients with predictably high risks of non-responsiveness to the first IVIG treatment. The second-line treatment is a second dose of IVIGs at the same dosage. Corticosteroids are the first complementary drug to IVIGs and the second-line treatment of choice. In case of further resistance to treatment, other therapeutic options include biological drugs, proposed as a third-line treatment. According to different guidelines, they include TNF blockers (infliximab, etanercept), anakinra, and interleukin-1 receptor antagonists. There are no clear data on the superiority of one of these drugs. Canakinumab is mentioned only in Italian guidelines; on the other hand, urinary trypsin inhibitor (ulinastatin) is mentioned in Japanese and Italian guidelines. Other therapeutic options in some guidelines include cyclosporine, methotrexate, and plasmapheresis, but they are reserved for patients resistant to the previously mentioned treatments [6,9,10,11].

Formation of aneurysms or ectasia occurs mainly in the coronary arteries, while scientific publications contain reports of changes, including among others, cerebral arteries [5,12]. Coronary artery aneurysms occur in 15% to 25% of untreated children and may lead to myocardial infarction, sudden cardiac death, or ischemic heart disease [4,13]. As it is known from the literature, there are also late complications of Kawasaki disease, such as stenosis or thrombosis of the coronary arteries [14] and calcified coronary artery aneurysms [15,16,17,18].

There are known scores for the development of coronary aneurysms and IVIG refractory cases. The Gunma scoring system is a risk scoring system often used to predict the severity of KD and coronary artery abnormalities, as well as for identifying IVIG non-responders among KD patients. [19]. Suzuki et al. recently reported the usefulness of an electrophysiological index calculated from the resting ECG recording, which allows the effective prediction of the subsequent development of coronary artery aneurysms in children with Kawasaki disease [20].

### Case Presentation

**Figure 1:**
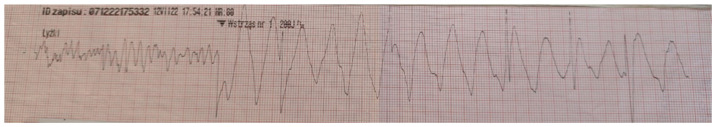
Electrocardiogram obtained from the emergency medical team with ventricular fibrillation recorded during sudden cardiac arrest.

A previously healthy 17-year-old boy was admitted to hospital after an episode of sudden cardiac arrest caused by ventricular fibrillation after successful cardiopulmonary resuscitation (Figure 1).

The incident occurred during a football game in the emergency care for children and adolescents where the boy was a patient. It was known from medical history that the boy was only under psychiatric outpatient clinic care due to moderate mental retardation and was constantly taking sodium valproate, risperidone, and clonidine. According to information obtained from the care centre’s director, the patient did not have any significant medical events in the past. The first troponin level 1.5 h after cardiac arrest was 156 ng/L, and the maximum (1559 ng/L) was recorded 14 h after the incident (troponin level norm < 11 ng/L). The other laboratory tests performed in the emergency department did not reveal any significant abnormalities that could indicate the cause of sudden cardiac arrest, the narcotic multi-test gave a negative result, and the ECG showed no changes that could indicate electrical heart disease.

**Figure 2:**
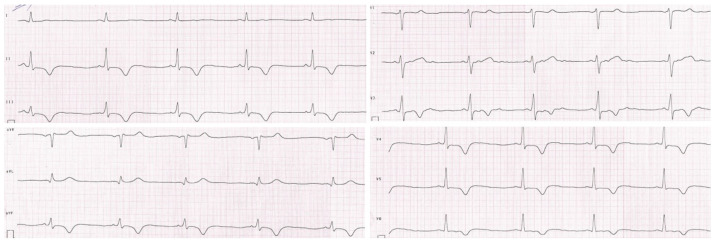
Resting electrocardiogram of the patient. Repolarization disturbances such as negative T waves in II, III, aVF, and V3–V6 leads and a positive T wave in the aVR lead.

The patient was initially hospitalized in the intensive care unit, where a cardiology consultation was carried out. Echocardiographic examination revealed segmental hypokinesis of the left ventricle, especially in the apical part, with preserved global contractility. Magnetic resonance imaging of the heart showed the presence of post-ischemic myocardial lesions. The resting ECG and 24 h Holter ECG recording revealed repolarization disturbances such as negative T waves in II, III, aVF, and V3–V6 leads, and a positive T wave in the aVR lead. Periodically, changes resembling delta waves were also observed in II, III, aVF, and V4 leads without other signs of pre-excitation (Figure 2).

Electrophysiological examination, performed at the Electrophysiology Clinic of the Central Clinical Hospital in Łódź, excluded the presence of an additional conduction pathway and did not trigger any arrhythmia; however, the scan revealed the presence of a calcified structure associated with the heart. Diagnoses were expanded to include coronary angiography, which showed the presence of most likely ruptured, clotted, well-calcified aneurysm of the left anterior descending artery (LAD) not filled with contrast, with collateral circulation (Video S1). In the right coronary artery (RCA), no aneurysmatic changes were found.

Video S1 in Supplementary Materials is a video of the coronary angiogram showing most likely ruptured, clotted, well-calcified aneurysm of the left anterior descending artery (LAD). RAO 25.7 degrees; caudal view of the chest, 0.8 degrees.

**Figure 3:**
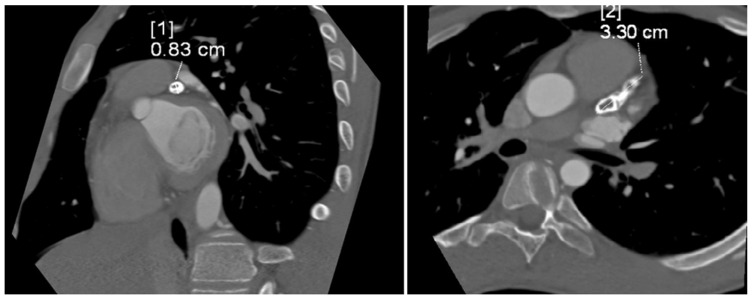
Computed tomography angiogram showing tubular, highly calcified, left coronary aneurysm (8.3 × 15 × 33 mm) without any features of contrasted lumen. Diameter [1]—8.3 mm; diameter [2]—33 mm.

The tubular, highly calcified structure, without any features of contrasted lumen, measuring 8.3 × 15 × 33 mm, was confirmed by CT angiogram of the coronary vessels (Figure 3). Relating the dimension obtained from imaging studies to the Z-score classification of coronary artery abnormalities, we obtained a Z-score of +11.2, which, together with an absolute dimension ≥ 8 mm according to the guidelines, corresponded to a giant aneurysm.

Based on the obtained test results, the mother’s interview was extended. She noted a severe infection in the boy in early infancy, with a fever lasting over a week, and a rash and oral lesions, which did not respond to antipyretic and antibiotic therapies. No one suspected Kawasaki disease at that time. According to the mother, he was diagnosed with a severe form of scarlet fever. Based on this interview, the presumption of undiagnosed Kawasaki disease in infancy was made, complicated in this case by the giant aneurysm of the left coronary artery.

The cardiac arrest occurred as a result of ventricular fibrillation, most likely induced by myocardial ischaemia during exertion. Due to the normal ejection fraction in follow-up echocardiography and well-developed collateral circulation, the patient did not qualify for an interventional cardiology procedure or cardiac surgery. As a secondary prevention of sudden cardiac arrest, the boy was implanted with a subcutaneous cardioverter-defibrillator. The patient remains asymptomatic and under further cardiological care.

## 2. Discussion

In patients with a history of Kawasaki disease, the main consequence may be involvement of the coronary arteries, especially in those without treatment. It is estimated that large doses of intravenous immunoglobulins reduce the prevalence of coronary artery aneurysms to approximately 5% [21].

Patients with coronary artery aneurysms can be completely asymptomatic. When symptomatic, chest pain, dyspnoea, easy fatigue, acute coronary ischaemia/acute myocardial infarction, or acute heart failure or arrhythmia can occur. The first symptom can also be sudden cardiac death [22,23,24,25].

Ayusawa et al. investigated the incidence of sudden cardiac deaths in students with a history of Kawasaki disease. Sudden cardiac death occurred in 10 students who suffered from Kawasaki disease in childhood, complicated by a coronary artery aneurysm. The same authors stated that the cause of death in two patients was an arrhythmic cardiac arrest event [22]. Intense physical exercise can be a trigger of symptoms in a patient with lesions in coronary vessels. Burns et al. in a retrospective study reviewed 74 cases to characterise the late cardiac sequelae of Kawasaki disease. In 82% of patients, symptoms such as chest pain, dyspnoea, or arrhythmia occurred, as in our patient, during intense physical exercise. A total of 18 patients died due to cardiovascular complications after presumed Kawasaki disease, of which 13 deaths were exercise-induced. Angiographic findings confirmed the presence of aneurysms in 93.2% of cases, with complete vessel occlusion in 66.1% of cases. Aneurysms most often involved the right coronary artery (RCA)—61.5%. LAD changes were described in 53.8% of cases [24]. In reports of coronary artery aneurysms in adults, the right coronary artery is most commonly involved, and the aetiological factor is atherosclerosis [26,27]. According to the 2020 guidelines for the diagnosis and management of patients with cardiovascular sequelae in Kawasaki disease, the place where the presence of aneurysms is most frequently found is the proximal fragment of the LAD [6]. In our patient, the aneurysm was present in the left anterior descending artery.

The consequence of undiagnosed and untreated Kawasaki disease may be symptoms imitating myocarditis or myocardial ischaemia. Uzdavinyte Gateliene et al. reported the case of a 25-year-old woman who presented to hospital with chest pain and dyspnoea while skiing. She was initially diagnosed with acute myocarditis. However, symptoms recurred after 6 months, related to minimal physical activity. The diagnostics were extended, based on which a 10 mm LAD aneurysm and a 7 mm RCA aneurysm were diagnosed, probably after Kawasaki disease at the age of 5 years [28]. In turn, Sliem et al. described the case of a 29-year-old patient whose first symptom of Kawasaki disease was sudden cardiac arrest during a birthday party. The previously healthy woman was successfully resuscitated. Her coronary angiogram revealed severe coronary artery disease with multiple ulcerations, aneurysms, and occlusions [29].

As was mentioned before, sudden cardiac death may be the first manifestation of complications of Kawasaki disease. Such a case was described by Zhang et al., where a 5-year-old boy died on the eighth day of the acute phase of Kawasaki disease as a result of the rupture of a giant left anterior descending coronary artery aneurysm, which was confirmed at autopsy. The child was diagnosed with mumps and scarlet fever was also suspected. The boy received ribaviryn and cefixim. The patient did not receive treatment typical for the acute phase of KD during 8 days of hospitalization. According to the literature, rupture of a coronary aneurysm occurs very rarely within the first few months after KD and in most cases, it concerns, as in this case, giant aneurysms [30,31]. Our patient also did not receive a diagnosis of KD during the acute symptoms, nor did he receive treatment that could have prevented the formation of a giant aneurysm. As in the case mentioned above, a severe form of scarlet fever was initially diagnosed.

We need to remember that clinical diagnosis of Kawasaki disease can be difficult, even with a doctor’s sufficient knowledge and clinical experience. Early clinical diagnosis may be difficult when the diagnosis criteria are not met for failure to diagnose Kawasaki disease. Therefore, sometimes the diagnosis is delayed or the disease is diagnosed as a different one. The differential diagnosis of Kawasaki disease should include infectious diseases (measles, scarlet fever, Epstein–Barr, adeno- and enterovirus infections, staphylococcal scales skin syndrome), toxic shock syndrome, leptospirosis, Yersinia infection, allergic reactions (drug hypersensitivity reactions, Stevens–Johnson syndrome), Rocky Mountain spotted fever, juvenile chronic arthritis, polyarteritis nodosa, arthritis, and mercury hypersensitivity reaction [2,4]. In all patients with fever lasting over 5 days, especially unresponsive to antipyretic and antibiotic therapies, KD should be suspected.

A summary of a few case reports on cardiac arrest and/or sudden cardiac death as a consequence of undiagnosed Kawasaki disease is presented in Table 1.

During diagnostics, after our patient’s cardiac arrest, based on available data from the interview, physical examination, and basic diagnostic tests, we initially looked for channelopathy like QT prolongation syndrome, Brugada syndrome, or CPVT, as well as other arrhythmogenic conditions that increase the risk for sudden cardiac arrest during intense physical exercise in adolescents, such as muscular dystrophies or a structural heart defect. Only coronary angiography revealed a calcified aneurysm of the left anterior descending coronary artery, which allowed us to conclude that the patient had undiagnosed Kawasaki disease in early childhood. Treatment of patients with KD who develop myocardial ischaemia or myocardial infarction due to coronary artery lesions requires percutaneous coronary intervention or coronary artery bypass grafting (CABG). However, coronary artery lesions can differ from atherosclerotic ones. Therefore, careful attention should be paid to aneurysms or calcified lesions. CABG should be considered first for patients with acute coronary syndrome [6,10]. Management with paediatric patients often requires an individual approach to the patient. In our case, the boy had one aneurysm and well-developed collateral circulation. Myocardial ischaemia most likely was induced by intense exertion, and cardiac arrest occurred as a result of ventricular fibrillation. During a consultation between cardiologists and a cardiac surgeon, it was determined that in this case, implantation of a cardioverter-defibrillator for secondary prevention of sudden cardiac death would be the optimal solution.

## 3. Conclusions

Our case concerns a patient with undiagnosed and untreated Kawasaki disease in infancy, complicated by a ruptured aneurysm of the left coronary artery in the past and sudden cardiac arrest during intense physical activity due to ventricular fibrillation, with return of spontaneous circulation after defibrillation.

In the differential diagnosis of sudden cardiac arrest, especially in young patients, it seems reasonable to consider the complications of Kawasaki disease. In turn, patients diagnosed and treated with Kawasaki disease should undergo cardiological screening in accordance with guidelines to detect and avoid potential complications. However, we must remember that undiagnosed Kawasaki disease may remain undetected for a long time and lead to serious and life-threatening complications such as cardiac arrest.

## Figures and Tables

**Table 1:** A summary of a few case reports on cardiac arrest and/or sudden cardiac death as a consequence of undiagnosed Kawasaki disease.

Authors, Date of Publication	Sex, Age	Initial Diagnosis	Coronary Artery Involvement	Follow-Up
Uzdavinyte Gateliene et al., 2023 [28]	F, 25 years old	myocarditis	LAD, RCA	survived
Sliem et al., 2023 [29]	F, 29 years old	myocardial infarction	LAD	survived
Zhang et al., 2018 [30]	M, 5 years old	mumps and scarlet fever	LAD	died
Stahl et al., 2019 [32]	M, 14 years old	cardiac arrest	LAD, RCA	survived
Zhu et al., 2019 [33]	M, 29 years old	cardiac arrest, embolism	LAD, RCA	survived
Motta et al., 2024 [34]	M, 17 years old	cardiac arrest	LMCA, RCA	survived

F—female, M—male, LAD—left anterior descending artery, RCA—right coronary artery, LMCA—left main coronary artery.

## Data Availability

The data used to support the findings of this study are included within the article.

## Supplementary Materials

The following supporting information can be downloaded at: https://www.mdpi.com/article/10.3390/jcm13216380/s1, Video S1: Video of the coronary angiogram showing most likely ruptured, clotted, well-calcified aneurysm of the left anterior descend-ing artery (LAD). RAO 25.7 degrees, caudal view of the chest 0.8 degrees.
